# Author Correction: VAMPnets for deep learning of molecular kinetics

**DOI:** 10.1038/s41467-018-06999-0

**Published:** 2018-10-22

**Authors:** Andreas Mardt, Luca Pasquali, Hao Wu, Frank Noé

**Affiliations:** Department of Mathematics and Computer Science, Freie Universität Berlin, Arnimallee 6, 14195 Berlin, Germany

Correction to: *Nature Communications* ; 10.1038/s41467-017-02388-1; published online 02 Jan 2018

In the original version of this Article, financial support was not fully acknowledged. The PDF and HTML versions of the Article have now been corrected to include funding from the Deutsche Forschungsgemeinschaft Grant SFB958/A04.

The original article can be found online at 10.1038/s41467-017-02388-1.

