# Supplementary material for: VAMPnets for deep learning of molecular kinetics
Source: Nat Commun. 2018 Jan 2;9:5. doi: 10.1038/s41467-017-02388-1 (PMC5750224; doi:10.1038/s41467-017-02388-1)
Supplement: Supplementary file 1 — Supplementary Information [file 41467_2017_2388_MOESM1_ESM.pdf]

Supplementary Information

Supplementary Note 1

Here we define the mean-free feature transformations

$$\bar{\boldsymbol{\chi}}_0 = \boldsymbol{\chi}_0 - \mathbb{E}_t\left[\boldsymbol{\chi}_0\left(\mathbf{x}_t\right)\right], \quad \bar{\boldsymbol{\chi}}_1 = \boldsymbol{\chi}_1 - \mathbb{E}_{t+\tau}\left[\boldsymbol{\chi}_1\left(\mathbf{x}_{t+\tau}\right)\right].$$

The subspace VAMP-2 score is invariant with respect to linear transformations of basis functions, therefore

$$\begin{aligned}
\hat{R}_2\left[\begin{pmatrix}\mathbb{1}\\ \boldsymbol{\chi}_0\end{pmatrix}, \begin{pmatrix}\mathbb{1}\\ \boldsymbol{\chi}_1\end{pmatrix}\right] &= \hat{R}_2\left[\begin{pmatrix}\mathbb{1}\\ \bar{\boldsymbol{\chi}}_0\end{pmatrix}, \begin{pmatrix}\mathbb{1}\\ \bar{\boldsymbol{\chi}}_1\end{pmatrix}\right] \\
&= \left\|\begin{bmatrix}1 & \\ & \bar{\mathbf{C}}_{00}\end{bmatrix}^{-\frac{1}{2}}\begin{bmatrix}1 & \\ & \bar{\mathbf{C}}_{01}\end{bmatrix}\begin{bmatrix}1 & \\ & \bar{\mathbf{C}}_{11}\end{bmatrix}^{-\frac{1}{2}}\right\|_F^2 \\
&= \left\|\bar{\mathbf{C}}_{00}^{-\frac{1}{2}}\bar{\mathbf{C}}_{01}\bar{\mathbf{C}}_{11}^{-\frac{1}{2}}\right\|_F^2 + 1
\end{aligned}$$

and

$$\mathrm{d}\hat{R}_2\left[\begin{pmatrix}\mathbb{1}\\ \boldsymbol{\chi}_0\end{pmatrix}, \begin{pmatrix}\mathbb{1}\\ \boldsymbol{\chi}_1\end{pmatrix}\right] = \mathrm{trace}\left(\mathrm{d}\left(\bar{\mathbf{C}}_{01}\bar{\mathbf{C}}_{11}^{-1}\bar{\mathbf{C}}_{01}^{\top}\mathbf{C}_{00}^{-1}\right)\right).$$

Substituting Eq. (7-9) into the above equation leads to

$$\begin{aligned}
\mathrm{d}\hat{R}_2 = &\frac{2}{T-1}\mathrm{tr}\left(\bar{\mathbf{C}}_{00}^{-1}\bar{\mathbf{C}}_{01}\bar{\mathbf{C}}_{11}^{-1}\left(\bar{\mathbf{Y}} - \bar{\mathbf{C}}_{01}^{\top}\bar{\mathbf{C}}_{00}^{-1}\bar{\mathbf{X}}\right)\mathrm{d}\mathbf{X}^{\top}\right) \\
&+ \frac{2}{T-1}\mathrm{tr}\left(\bar{\mathbf{C}}_{11}^{-1}\bar{\mathbf{C}}_{01}^{\top}\bar{\mathbf{C}}_{00}^{-1}\left(\bar{\mathbf{X}} - \bar{\mathbf{C}}_{01}\bar{\mathbf{C}}_{11}^{-1}\bar{\mathbf{Y}}\right)\mathrm{d}\mathbf{Y}^{\top}\right),
\end{aligned}$$

where tr is the matrix trace. Therefore,

$$\begin{aligned}
\nabla_{\mathbf{X}}\hat{R}_2 &= \frac{2}{m-1}\bar{\mathbf{C}}_{00}^{-1}\bar{\mathbf{C}}_{01}\bar{\mathbf{C}}_{11}^{-1}\left(\bar{\mathbf{Y}} - \bar{\mathbf{C}}_{01}^{\top}\bar{\mathbf{C}}_{00}^{-1}\bar{\mathbf{X}}\right) \\
\nabla_{\mathbf{Y}}\hat{R}_2 &= \frac{2}{m-1}\bar{\mathbf{C}}_{11}^{-1}\bar{\mathbf{C}}_{01}^{\top}\bar{\mathbf{C}}_{00}^{-1}\left(\bar{\mathbf{X}} - \bar{\mathbf{C}}_{01}\bar{\mathbf{C}}_{11}^{-1}\bar{\mathbf{Y}}\right)
\end{aligned}$$

SUPPLEMENTARY FIGURES

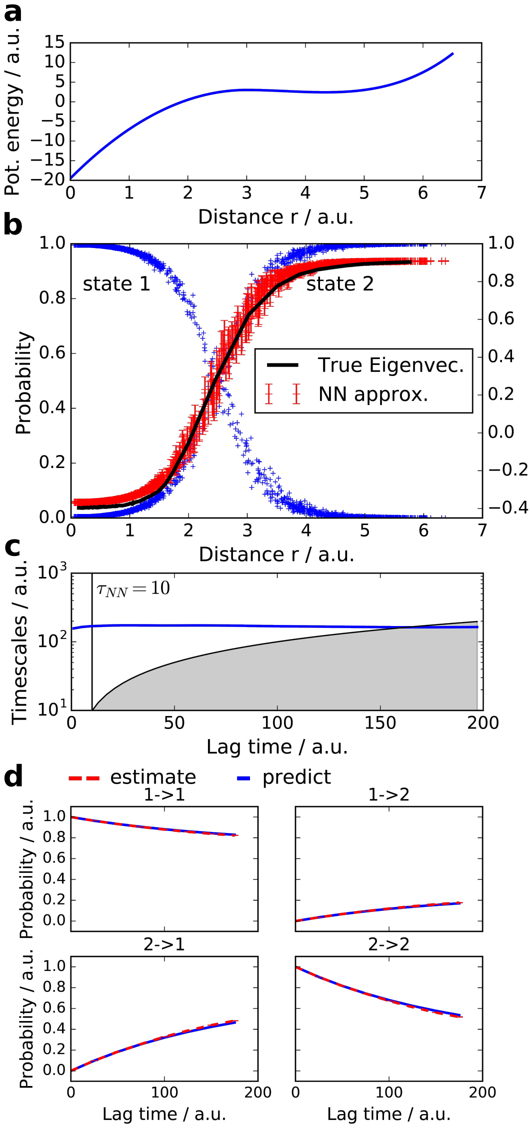

Supplementary Figure 1: VAMPnet model of a simple protein folding model. (**a**) Potential energy as a function of the radial position r. (**b**) Eigenvector of the slowest process calculated by direct numerical approximation on the radial position r (black) and approximated by the neural network build on the 5D-coordinates with five output nodes with $\tau = 10$ (red). Reported here is the mean and one standard deviation for the neural network over 100 runs. Activation of the two Softmax output nodes define the state membership probabilities (blue). (**c**) Relaxation timescales computed from the Koopman model using the neural network transformation. (**d**) Chapman-Kolmogorov test comparing long-time predictions of the Koopman model estimated at $\tau = 10$ and estimates at longer lag times.

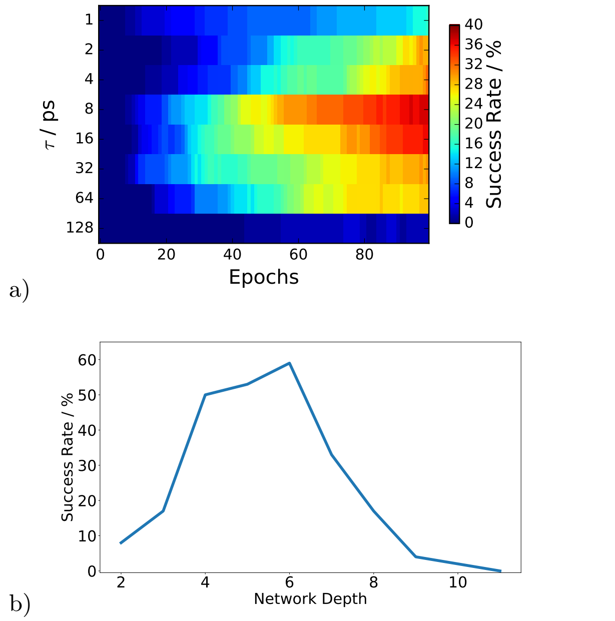

Supplementary Figure 2: Training success rate in alanine dipeptide VAMPnets with six output states as a function of hyperparameters. Training success rate is defined as the fraction of optimizations of the network to find all three slow processes (see text for details). (a) Training success rate in a five-layer network as a function of the lag time τ and the number of optimization epochs. (b) Training success rate at lag time $\tau = 8$ ps and after 100 epochs as a function of the network depth.

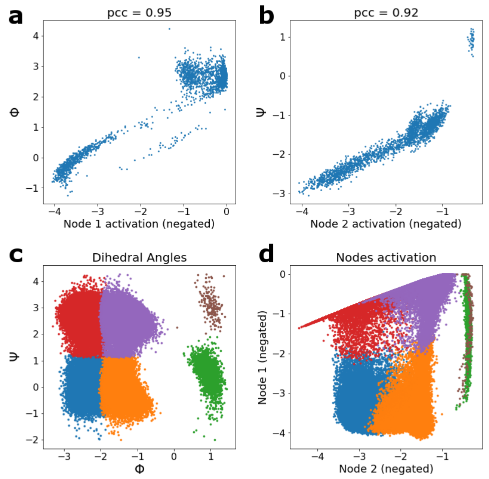

Supplementary Figure 3: Network with a bottleneck of two nodes implicitly learns the transformation from heavy atom positions ϕ and ψ torsion angles. (**a, b**): Correlation of torsion angles and the activations of the bottleneck nodes. Pearson correlation coefficient (pcc) is shown above. (**c**) Samples plotted as a function of ϕ and ψ angles, colored according to six clusters found by a optimized network with six output state and no bottleneck. (**d**) Samples with same colors, plotted on the activations of the bottleneck nodes.
